# Real-world outcomes of ponatinib treatment in 724 patients with CML and Ph^+^ ALL: a post-marketing surveillance study with a special interest in arterial occlusive events in Japan

**DOI:** 10.1093/jjco/hyae061

**Published:** 2024-05-15

**Authors:** Naoto Takahashi, Takeshi Kondo, Yuji Ikari, Yoshihiro Fukumoto, Kiyohiko Hatake, Akira Masunari, Seiji Nishibayashi, Akiko Kageyama, Yasuhiko Fukuta, Arinobu Tojo

**Affiliations:** Department of Hematology, Nephrology and Rheumatology, Akita University Graduate School of Medicine, Akita, Japan; Department of Hematology, Blood Disorders Center, Aiiku Hospital, Sapporo, Japan; Department of Cardiology, Tokai University Hospital, Isehara, Japan; Division of Cardiovascular Medicine, Department of Internal Medicine, Kurume University School of Medicine, Kurume, Japan; Department of Lymphoma/Hematology Center, Mita Hospital, International University of Health and Welfare, Tokyo, Japan; Otsuka Pharmaceutical Co., Ltd, Tokyo; Otsuka Pharmaceutical Co., Ltd, Tokyo; Otsuka Pharmaceutical Co., Ltd, Tokyo; Otsuka Pharmaceutical Co., Ltd, Tokyo; Tokyo Medical and Dental University, Tokyo, Japan

**Keywords:** acute lymphoblastic leukemia, arterial occlusive events, chronic myeloid leukemia, ponatinib, post-marketing surveillance

## Abstract

**Background:**

In September 2016, ponatinib was approved in Japan for the treatment of patients with chronic myeloid leukemia with resistance/intolerance to prior tyrosine kinase inhibitors and patients with relapsed or refractory Philadelphia chromosome-positive acute lymphoblastic leukemia.

**Methods:**

We conducted a post-marketing all-case surveillance to study the safety and efficacy of ponatinib in clinical practice, focusing on arterial occlusive events.

**Results:**

Data from 724 patients were collected for 2 years from the initiation of ponatinib. The arterial occlusive events were reported in 6.49% (47/724) with an exposure-adjusted incidence rate of 6.8/100 person-years. The risks associated with arterial occlusive events were age and comorbidities including hypertension and diabetes. At 104 weeks, the cumulative major molecular response rate in patients with chronic-phase chronic myeloid leukemia was 67.2% and the complete cytogenetic response in patients with Philadelphia chromosome-positive acute lymphoblastic leukemia was 80.0%. Furthermore, the estimated 1-year overall survival rate was 98.5% for chronic-phase chronic myeloid leukemia and 68.6% for Philadelphia chromosome-positive acute lymphoblastic leukemia.

**Conclusions:**

This surveillance demonstrated that ponatinib has a favorable safety and efficacy profile in Japanese patients and also showed the necessity of closely monitoring arterial occlusive events in older adults and patients with predisposing factors for atherosclerosis.

## Teaser

Ponatinib has a favorable safety and efficacy profile in Japanese patients: close monitoring of arterial occlusive events is indispensable among high-risk patients.

## Introduction

The development of tyrosine kinase inhibitors (TKIs) for the treatment of chronic myeloid leukemia (CML) or Philadelphia chromosome-positive acute lymphoblastic leukemia (Ph^+^ ALL) has led to the introduction of a selective targeted therapy that inhibits the BCR::ABL1 tyrosine kinase and to an improvement in treatment outcomes by suppressing the growth of leukemia cells (1). However, patients may be intolerant to TKIs due to adverse events (AEs) (2) or may develop resistance to TKIs due to mutations in the ABL kinase domain (3).

Ponatinib, a third-generation TKI, was approved in Japan by the Ministry of Health, Labour and Welfare in September 2016 for the treatment of patients with CML with resistance/intolerance to prior TKIs and patients with relapsed and refractory Ph^+^ ALL, based on the results from the phase 2 Ponatinib Ph^+^ ALL and CML Evaluation (PACE) trial conducted outside Japan (4) and a phase 1/2 trial conducted in Japan (5).

Ponatinib has inhibitory activity against BCR::ABL1 kinase, including a variant with threonine-to-isoleucine at position 315 (T315I) (4) but is associated with a higher risk of major arterial events than other TKIs among patients with CML (6). In the phase 2 PACE trial, the incidence of arterial occlusive events (AOEs, i.e. vascular ischemia or thrombosis) was 25%, and the exposure-adjusted incidence rate of AOEs per 100 person-years was 13.8 (7). In the phase 1/2 trial of Japanese patients, 14% (5/35) of patients treated with ponatinib experienced AOEs (5).

Since the number of Japanese patients treated with ponatinib in clinical trials so far is quite limited, the safety and efficacy of ponatinib in Japanese patients require further evaluation. In addition, findings from randomized clinical trials, which are conducted in carefully selected patients and follow strictly controlled drug regimens, cannot always be generalized to the whole population (8). Observational studies conducted outside Japan to explore real-life experience with ponatinib have provided valuable information that is sometimes different from that obtained in the clinical trial cohorts (9–11). Therefore, we conducted a post-marketing surveillance to study the safety and efficacy of ponatinib, focusing on AOEs, to optimize its use in clinical practice in Japan.

## Methods

### Surveillance design and patients

This was a post-marketing surveillance to obtain real-world data on ponatinib in Japanese patients with CML with resistance/intolerance to prior kinase inhibitors and patients with relapsed/refractory Ph^+^ ALL. The surveillance was conducted in accordance with the Japanese regulatory requirements stipulated in Good Post-Marketing Study Practice. Informed consent was obtained from all patients who were to receive ponatinib in accordance with the package insert of ponatinib, but under these regulations, central Institutional Review Board oversight and patient consent are not required.

The case report forms were collected between 21 November 2016 and 30 June 2018. The observation period was 2 years or until the end of ponatinib administration, and the forms were collected at 3 months, 6 months, 1 year,and 2 years after the initiation of ponatinib. Information collected was as follows: patient characteristics, previous treatment history, administration status of ponatinib, safety (e.g. AEs and laboratory data) and efficacy information (e.g. hematological findings and cytogenetic/molecular analyses).

The recommended starting dose of ponatinib was 45 mg once daily, which might be adjusted to a lower dose (30 or 15 mg) based on the condition of each patient.

### Safety analysis

We collected information on AEs to assess the safety of ponatinib. A serious adverse reaction (ADR) is an AE that requires hospitalization or prolongation of existing hospitalization, causes a congenital malformation, results in persistent or significant disability or incapacity, is life-threatening, or results in death (12).

This surveillance focused on the real-world incidence of AOEs in Japanese patients treated with ponatinib. AEs were coded according to the Medical Dictionary for Regulatory Activities/J (MedDRA/J), version 25.0, and AOEs were categorized based on preferred terms related to vascular ischemia or thrombosis. An exposure-adjusted incidence rate of AOEs was calculated as (the number of first events during observation)/(the total exposure during observation, person-years) × 100 (7). We also analyzed the association between AOEs and patient characteristics, such as age, history of ischemic disease and specific comorbidities (diabetes, hypertension and dyslipidemia).

### Efficacy analysis: Cytogenic and molecular responses

The cytogenetic response was assessed by calculating the ratio of the number of Ph^+^ cells to the number of observed cells in bone marrow samples and was classified into the following categories: complete cytogenetic response (CCyR, Ph^+^ cells 0%), partial cytogenetic response (PCyR, Ph^+^ cells 1% to 35%) and others (13). The molecular response was determined according to the international standard (IS) for the level of *BCR::ABL1* transcripts as assessed by quantitative Reverse Transcription-PCR (RT-PCR) assay and was classified into the following categories: major molecular response (MMR; *BCR::ABL1* transcripts [IS] ≤ 0.1%), MR4.0 (*BCR::ABL1* transcripts [IS] ≤ 0.01%), MR4.5 (*BCR::ABL1* transcripts [IS] ≤ 0.0032%) and non-MMR (*BCR::ABL1* transcripts [IS] > 0.1%) (13,14).

### Statistical analysis

Statistical analysis was performed using SAS software, Version 9.3 (SAS Institute Japan Ltd., Japan). The number of patients, the mean and standard deviation (SD), or the median and range were calculated as summary statistics. Univariable logistic regression analysis evaluated the association of patient characteristics (sex, age, performance status, Philadelphia chromosome, *BCR::ABL1* transcripts, *BCR::ABL1* mutations, smoking, comorbidities, history of ischemic disease, average daily dose of ponatinib, starting daily dose of ponatinib and maximum daily dose of ponatinib) with ADRs and AOEs to obtain the odds ratio (OR) and its 95% confidence interval (CI). The cumulative incidence of AOEs, cumulative response rates and survival rates were shown in Kaplan–Meier plots.

## Results

### Patient characteristics

A total of 807 patients were enrolled at 334 facilities (Supplementary Fig. 1). Case report forms were collected from 724 patients. The safety analysis was conducted in all 724 patients, and 116 patients were excluded from the efficacy analysis for the following reasons: (i) they were treated for diseases other than CML or Ph^+^ ALL (*n* = 6), (ii) they were not evaluated for all efficacy endpoints (*n* = 104) and/or (iii) they had received drugs with the same active ingredient composition as ponatinib before the surveillance (*n* = 10).

Of the 724 enrolled patients, 58.1% (421/724) were males, and the median (range) age was 62.0 (4–94) years (Table 1). The median (range) duration of disease was 373.0 (1–8726) days for the overall population, 1350.0 (1–8726) days for patients with CML-CP and 222.0 (1–6031) days for patients with Ph^+^ ALL. The performance status (PS) was 0 in 48.8% (353/724) of patients, 1 in 35.4% (256/724), 2 in 10.2% (74/724) and 3 to 4 in 5.2% (38/724). Comorbidities were present in 68.0% (492/724) of patients, including hypertension in 26.8% (194/724), diabetes in 20.2% (146/724) and dyslipidemia in 19.9% (144/724). Mutations in *BCR::ABL1* were tested in 61.3% (444/724) and were positive in 235/444, with 171/444 having the T315I mutation. The number of prior TKIs was 0 in 2.6% (19/724) of patients, 1 in 48.5% (351/724), 2 in 30.7% (222/724) and 3 or 4 in 17.7% (128/724). Combination chemotherapy was used in 40.6% (294/724) of patients: 6.7% (13/193) in CML-CP, 21.9% (7/32) in CML-AP, 45.6% (47/103) in CML-BC and 56.9% (222/390) in Ph^+^ ALL.

**Table 1:** Baseline characteristics of patients

	Overall	CML-CP	CML-AP	CML-BC	Ph^+^ ALL	Others^a^
Characteristics	*N* = 724	*N* = 193	*N* = 32	*N* = 103	*N* = 390	*N* = 6
Male	421 (58.1)	121 (62.7)	26 (81.3)	67 (65.0)	205 (52.6)	2 (33.3)
Age, years	62.0 (4–94)	61.0 (9–94)	63.5 (21–86)	57.0 (11–87)	63.0 (4–91)	57.0 (15–72)
Duration of disease, days	373.0 (1–8726)	1350.0 (1–8726)	729.0 (1–7331)	253.0 (1–6865)	222.0 (1–6031)	295.5 (19–1524)
Not recorded	18 (2.5)	8 (4.1)	1 (3.1)	4 (3.9)	5 (1.3)	0 (0.0)
Reason to start ponatinib^b^						
Resistance to TKIs	-	108 (56.0)	23 (71.9)	73 (70.9)	231 (59.2)	-
Intolerance to TKIs	-	62 (32.1)	7 (21.9)	17 (16.5)	60 (15.4)	-
Others	-	17 (8.8)	1 (3.1)	11 (10.7)	60 (15.4)	-
Performance status						
0	353 (48.8)	144 (74.6)	21 (65.6)	37 (35.9)	147 (37.7)	4 (66.7)
1	256 (35.4)	37 (19.2)	8 (25.0)	41 (39.8)	168 (43.1)	2 (33.3)
2	74 (10.2)	7 (3.6)	2 (6.3)	12 (11.7)	53 (13.6)	0 (0.0)
3	26 (3.6)	3 (1.6)	0 (0.0)	9 (8.7)	14 (3.6)	0 (0.0)
4	12 (1.7)	0 (0.0)	1 (3.1)	4 (3.9)	7 (1.8)	0 (0.0)
Not recorded	3 (0.4)	2 (1.0)	0 (0.0)	0 (0.0)	1 (0.3)	0 (0.0)
Comorbidities						
Yes	492 (68.0)	113 (58.5)	23 (71.9)	65 (63.1)	285 (73.1)	6 (100.0)
Hypertension	194 (26.8)	49 (25.4)	7 (21.9)	29 (28.2)	107 (27.4)	2 (33.3)
Diabetes	146 (20.2)	29 (15.0)	4 (12.5)	20 (19.4)	92 (23.6)	1 (16.7)
Dyslipidemia	144 (19.9)	28 (14.5)	9 (28.1)	17 (16.5)	88 (22.6)	2 (33.3)
Ischemic disease^c^	53 (7.3)	14 (7.3)	2 (6.3)	11 (10.7)	26 (6.7)	0 (0.0)
No	231 (31.9)	80 (41.5)	9 (28.1)	38 (36.9)	104 (26.7)	0 (0.0)
Not recorded	1 (0.1)	0 (0.0)	0 (0.0)	0 (0.0)	1 (0.3)	0 (0.0)
Smoking						
Yes	187 (25.8)	50 (25.9)	10 (31.3)	26 (25.2)	99 (25.4)	2 (33.3)
No	525 (72.5)	139 (72.0)	22 (68.8)	75 (72.8)	285 (73.1)	4 (66.7)
Not recorded	12 (1.7)	4 (2.1)	0 (0.0)	2 (1.9)	6 (1.5)	0 (0.0)
Number of prior TKIs						
0	19 (2.6)	0 (0.0)	0 (0.0)	2 (1.9)	16 (4.1)	1 (16.7)
1	351 (48.5)	41 (21.2)	8 (25.0)	51 (49.5)	246 (63.1)	5 (83.3)
2	222 (30.7)	69 (35.8)	8 (25.0)	30 (29.1)	115 (29.5)	0 (0.0)
3	81 (11.2)	49 (25.4)	7 (21.9)	16 (15.5)	9 (2.3)	0 (0.0)
4	47 (6.5)	34 (17.6)	9 (28.1)	4 (3.9)	0 (0.0)	0 (0.0)
Not recorded	4 (0.6)	0 (0.0)	0 (0.0)	0 (0.0)	4 (1.0)	0 (0.0)
*BCR::ABL1* mutations						
Tested	444 (61.3)	123 (63.7)	25 (78.1)	76 (73.8)	217 (55.6)	3 (50.0)
Positive	235 (32.5)	38 (19.7)	15 (46.9)	50 (48.5)	132 (33.8)	0 (0.0)
T315I mutation	171 (23.6)	23 (11.9)	8 (25.0)	31 (30.1)	109 (27.9)	0 (0.0)
Negative	209 (28.9)	85 (44.0)	10 (31.3)	26 (25.2)	85 (21.8)	3 (50.0)

Values are given as n (%) or median (range).

AP, accelerated-phase; BC, blast crisis; CML, chronic myeloid leukemia; CP, chronic-phase; Ph^+^ ALL, Philadelphia chromosome-positive acute lymphoblastic leukemia; TKI, tyrosine kinase inhibitor

^a^Others include Ph^+^ ALL intolerance to prior treatment (*n* = 1), acute myeloid leukemia with c-Kit mutations (*n* = 1), CML-CP without resistance/intolerance to prior TKIs (*n* = 1), Ph^+^ ALL without relapse or refractory disease (*n* = 2), Philadelphia chromosome-like acute lymphoblastic leukemia (*n* = 1).

^b^Patients with both TKI resistance and intolerance are included in TKI intolerance; patients with TKI resistance and other reasons are included in TKI resistance; patients with TKI intolerance and other reasons are included in TKI intolerance; and patients with TKI resistance, intolerance, and other reasons are included in TKI intolerance.

^c^Ischemic disease includes coronary artery disease, cerebrovascular disease, retinal artery occlusion, peripheral arterial occlusive disease, and venous thromboembolism.

### Summary of ponatinib administration

The median (range) follow-up duration was 258.0 (1–1436) days for the overall population, 729.5 (7–1436) days for patients with CML-CP and 168.0 (1–1254) days for patients with Ph^+^ ALL (Supplementary Table 1). The initial daily dose was 45 mg in 19.8% (143/724) of patients and 15 or 30 mg in 79.8% (578/724), and the maximum daily dose was 45 mg in 36.3% (263/724) of patients and 15 or 30 mg in 62.8% (455/724).

### Summary of adverse drug reactions

ADRs were observed in 58.29% (422/724) of patients (Table 2). The most common ADRs comprised platelet count decreased (8.15% [59/724]), hypertension (7.73% [56/724], rash (5.66% [41/724]), neutrophil count decreased (4.83% [35/724]) and hepatic function abnormal (4.28% [31/724]). The most common serious ADR was platelet count decreased (2.49% [18/724]).

**Table 2:** Summary of adverse drug reactions

Types of adverse drug	Overall		Any CML		CML-CP		CML-AP		CML-BC		Ph^+^ ALL		Others^a^	
reactions	N = 724		N = 328		N = 193		N = 32		N = 103		N = 390		N = 6	
Any adverse drug reaction	422 (58.29)		198 (60.37)		119 (61.66)		22 (68.75)		57 (55.34)		220 (56.41)		4 (66.67)	
														
Common adverse drug reactions in ≥3.0%	Overall	Serious	Overall	Serious	Overall	Serious	Overall	Serious	Overall	Serious	Overall	Serious	Overall	Serious
Vascular disorders														
Hypertension	56 (7.73)	5 (0.69)	33 (10.06)	2 (0.61)	22 (11.40)	2 (1.04)	4 (12.50)	0 (0.00)	7 (6.80)	0 (0.00)	23 (5.90)	3 (0.77)	0 (0.00)	0 (0.00)
Hepatobiliary disorders														
Hepatic function abnormal	31 (4.28)	5 (0.69)	12 (3.66)	1 (0.30)	8 (4.15)	0 (0.00)	0 (0.00)	0 (0.00)	4 (3.88)	1 (0.97)	18 (4.62)	4 (1.03)	1 (16.67)	0 (0.00)
Liver disorder	26 (3.59)	2 (0.28)	10 (3.05)	2 (0.61)	5 (2.59)	1 (0.52)	1 (3.13)	0 (0.00)	4 (3.88)	1 (0.97)	16 (4.10)	0 (0.00)	0 (0.00)	0 (0.00)
Skin and subcutaneous tissue disorders														
Rash	41 (5.66)	2 (0.28)	22 (6.71)	0 (0.00)	12 (6.22)	0 (0.00)	1 (3.13)	0 (0.00)	9 (8.74)	0 (0.00)	18 (4.62)	2 (0.51)	1 (16.67)	0 (0.00)
General disorders and administration site conditions														
Pyrexia	22 (3.04)	4 (0.55)	8 (2.44)	1 (0.30)	5 (2.59)	0 (0.00)	1 (3.13)	1 (3.13)	2 (1.94)	0 (0.00)	14 (3.59)	3 (0.77)	0 (0.00)	0 (0.00)
Investigations														
Neutrophil count decreased	35 (4.83)	7 (0.97)	14 (4.27)	1 (0.30)	3 (1.55)	0 (0.00)	4 (12.50)	1 (3.13)	7 (6.80)	0 (0.00)	20 (5.13)	6 (1.54)	1 (16.67)	0 (0.00)
Platelet count decreased	59 (8.15)	18 (2.49)	35 (10.67)	6 (1.83)	21 (10.88)	3 (1.55)	7 (21.88)	2 (6.25)	7 (6.80)	1 (0.97)	23 (5.90)	12 (3.08)	1 (16.67)	0 (0.00)
White blood cell count decreased	30 (4.14)	7 (0.97)	8 (2.44)	0 (0.00)	2 (1.04)	0 (0.00)	2 (6.25)	0 (0.00)	4 (3.88)	0 (0.00)	20 (5.13)	7 (1.79)	2 (33.33)	0 (0.00)

Values are given as n (%).

AP, accelerated-phase; BC, blast crisis; CML, chronic myeloid leukemia; CP, chronic-phase; Ph^+^ ALL, Philadelphia chromosome-positive acute lymphoblastic leukemia

^a^Others include Ph + ALL intolerance to prior treatment (n = 1), acute myeloid leukemia with c-Kit mutations (n = 1), CML-CP without resistance/intolerance to prior TKIs (n = 1), Ph + ALL without relapse or refractory disease (n = 2), Philadelphia chromosome-like acute lymphoblastic leukemia (n = 1).

Univariable logistic regression analysis found a significant association between ADRs and age (OR 1.017, 95% CI 1.008–1.026), hypertension (OR 1.701, 95% CI 1.203–2.403), diabetes (OR 1.478, 95% CI 1.012–2.160), dyslipidemia (OR 2.322, 95% CI 1.548–3.481), ischemic disease (OR 1.886, 95% CI 1.018–3.495), history of the ischemic disease (OR 1.672, 95% CI 1.056–2.647) and average daily dose of ponatinib (OR 0.974, 95% CI 0.960–0.988) (Supplementary Table 2).

### Cross intolerance between prior TKIs and ponatinib

Before this surveillance, imatinib had been used in 38.4% (278/724) of patients, dasatinib in 88.4% (640/724), nilotinib in 24.0% (174/724), bosutinib in 18.5% (134/724) and ponatinib (pre-market use) in 1.1% (8/724) (Supplementary Table 3). Cross intolerance between prior TKIs and ponatinib was observed in patients previously using imatinib (*n* = 18), dasatinib (*n* = 90), nilotinib (*n* = 10) and bosutinib (*n* = 29). Adverse events observed in common were as follows: platelet count decreased (imatinib, *n* = 1; dasatinib, *n* = 2), lipase increased (dasatinib, *n* = 1), pleural effusion (dasatinib, *n* = 1), rash (nilotinib, *n* = 1; bosutinib, *n* = 1), hepatic function abnormal (bosutinib, *n* = 1), leukoderma (bosutinib, *n* = 1), nausea (bosutinib, *n* = 1) and pancytopenia (bosutinib, *n* = 1).

### Arterial occlusive events and risk factors

The incidence of AOEs was 6.49% (47/724) in the overall population (Table 3), 6.22% (12/193) in patients with CML-CP and 5.90% (23/390) in patients with Ph^+^ ALL, and the exposure-adjusted incidence rate of AOEs per 100 person-years was 6.8, 4.5 and 7.1, respectively (Supplementary Table 4). The most common AOEs were cerebral infarction (1.52% [11/724]), disseminated intravascular coagulation (0.83% [6/724]) and myocardial infarction (0.69% [5/724]), and the most common serious AOEs were cerebral infarction (1.10% [8/724]) and myocardial infarction (0.69% [5/724]) (Table 3). AOEs leading to death during the follow-up duration were as follows: no AOEs leading to death in patients with CML-CP; myocardial infarction (*n* = 1) in accelerated-phase CML (CML-AP); myocardial infarction (*n* = 1) and thrombotic microangiopathy (*n* = 1) in blast crisis CML (CML-BC); and myocardial infarction (*n* = 2), disseminated intravascular coagulation (*n* = 2) and thrombotic microangiopathy (*n* = 1) in Ph^+^ ALL.

**Table 3:** Summary of arterial occlusive events (adverse drug reactions)

	Overall population (N = 724)		
Any arterial occlusive event	47 (6.49)		
Median treatment duration, days	266.0		
			
	Overall	Serious	Non-serious
Cardiac disorders			
Acute myocardial infarction	1 (0.14)	1 (0.14)	0 (0.00)
Angina pectoris	4 (0.55)	3 (0.41)	1 (0.14)
Angina unstable	2 (0.28)	2 (0.28)	0 (0.00)
Arteriospasm coronary	1 (0.14)	0 (0.00)	1 (0.14)
Coronary artery stenosis	1 (0.14)	1 (0.14)	0 (0.00)
Myocardial infarction	5 (0.69)	5 (0.69)^a^	0 (0.00)
Acute coronary syndrome	1 (0.14)	1 (0.14)	0 (0.00)
Cardiac discomfort	2 (0.28)	0 (0.00)	2 (0.28)
Stress cardiomyopathy	1 (0.14)	1 (0.14)	0 (0.00)
Nervous system disorders			
Cerebellar infarction	1 (0.14)	0 (0.00)	1 (0.14)
Cerebral artery embolism	1 (0.14)	0 (0.00)	1 (0.14)
Cerebral infarction	11 (1.52)	8 (1.10)	4 (0.55)
Transient ischemic attack	1 (0.14)	0 (0.00)	1 (0.14)
Lacunar infarction	1 (0.14)	0 (0.00)	1 (0.14)
Thalamic infarction	1 (0.14)	0 (0.00)	1 (0.14)
Thrombotic cerebral infarction	2 (0.28)	1 (0.14)	1 (0.14)
Vascular disorders			
Peripheral arterial occlusive disease	3 (0.41)	0 (0.00)	3 (0.41)
Arterial occlusive disease	1 (0.14)	0 (0.00)	1 (0.14)
Investigations			
Electrocardiogram ST segment depression	3 (0.41)	1 (0.14)	2 (0.28)
Electrocardiogram T wave inversion	1 (0.14)	1 (0.14)	0 (0.00)
Blood and lymphatic disorders			
Disseminated intravascular coagulation	6 (0.83)	2 (0.28)^b^	4 (0.55)
Thrombotic microangiopathy	3 (0.41)	3 (0.41)^c^	0 (0.00)

Values are given as n (%).

^a^Four patients died from myocardial infarction (CML-AP, n = 1; CML-BC, n = 1; Ph^+^ ALL, n = 2).

^b^Two patients died from disseminated intravascular coagulation (Ph^+^ ALL, n = 2).

^c^Two patients died from thrombotic microangiopathy (CML-BC, n = 1; Ph^+^ ALL, n = 1).

AP, accelerated-phase; BC, blast crisis; CML, chronic myeloid leukemia; CP, chronic-phase; Ph^+^ ALL, Philadelphia chromosome-positive acute lymphoblastic leukemia

**Figure 1:**
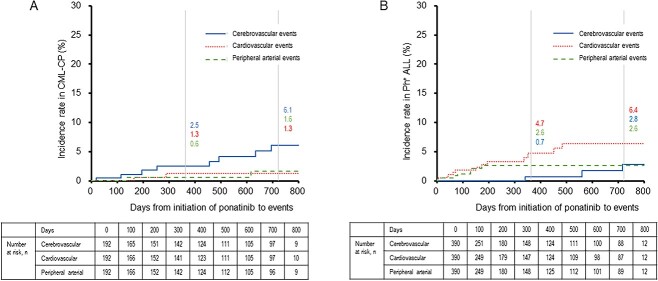
Cumulative incidence rates of arterial occlusive events in patients with CML-CP and Ph^+^ ALL. The cumulative incidence rates of cerebrovascular (Solid line), cardiovascular (Dotted line), and peripheral arterial events (Dashed line) are shown in patients with CML-CP (A) and Ph^+^ ALL (B). Incidence rates are labeled at years 1 and 2. CML, chronic myeloid leukemia; CP, chronic-phase; Ph^+^ ALL, Philadelphia chromosome-positive acute lymphoblastic leukemia.

The cumulative incidence rates of cerebrovascular, cardiovascular and peripheral arterial events were 2.5, 1.3 and 0.6% (after 1 year) and 6.1, 1.3 and 1.6% (after 2 years), respectively, in patients with CML-CP; 0.7, 4.7 and 2.6% (after 1 year) and 2.8, 6.4 and 2.6% (after 2 years), respectively, in patients with Ph^+^ ALL (Fig. 1).

Univariable logistic regression analysis revealed significant association between AOEs and age (OR 1.039, 95% CI 1.017–1.062), hypertension (OR 2.137, 95% CI 1.169–3.907) and diabetes (OR 1.950, 95% CI 1.026–3.707) (Table 4).

**Table 4:** Risks associated with arterial occlusive events, univariable logistic analysis

	Arterial occlusive events	
Factors	Odds ratio	95% CI
Female vs. male	0.633	0.337–1.191
Age	1.039	1.017–1.062^c^
Performance status	1.152	0.850–1.561
Philadelphia chromosome	0.555	0.161–1.192
*BCR::ABL1* transcripts	0.665	0.150–2.944
*BCR::ABL1* mutations	1.367	0.676–2.762
Smoking	0.960	0.487–1.891
Comorbidities		
Hypertension	2.137	1.169–3.907^c^
Diabetes	1.950	1.026-3.707^c^
Dyslipidemia	1.591	0.816-3.099
Ischemic disease^a^	1.558	0.589–4.119
Non-ischemic heart disease	-	-
History of ischemic disease^a^	1.400	0.632–3.098
Average daily dose of ponatinib^b^	0.990	0.962–1.019
Starting daily dose of ponatinib	0.979	0.952–1.006
Maximum daily dose of ponatinib	1.004	0.978–1.030

^a^Ischemic disease includes coronary artery disease, cerebrovascular disease, retinal artery occlusion, peripheral arterial occlusive disease, and venous thromboembolism.

^b^Average daily dose was calculated by dividing the total mass of ponatinib prescribed over the period by the study duration.

^c^95% CI of the corresponding odds ratio does not include 1.

### Efficacy of ponatinib

**Figure 2:**
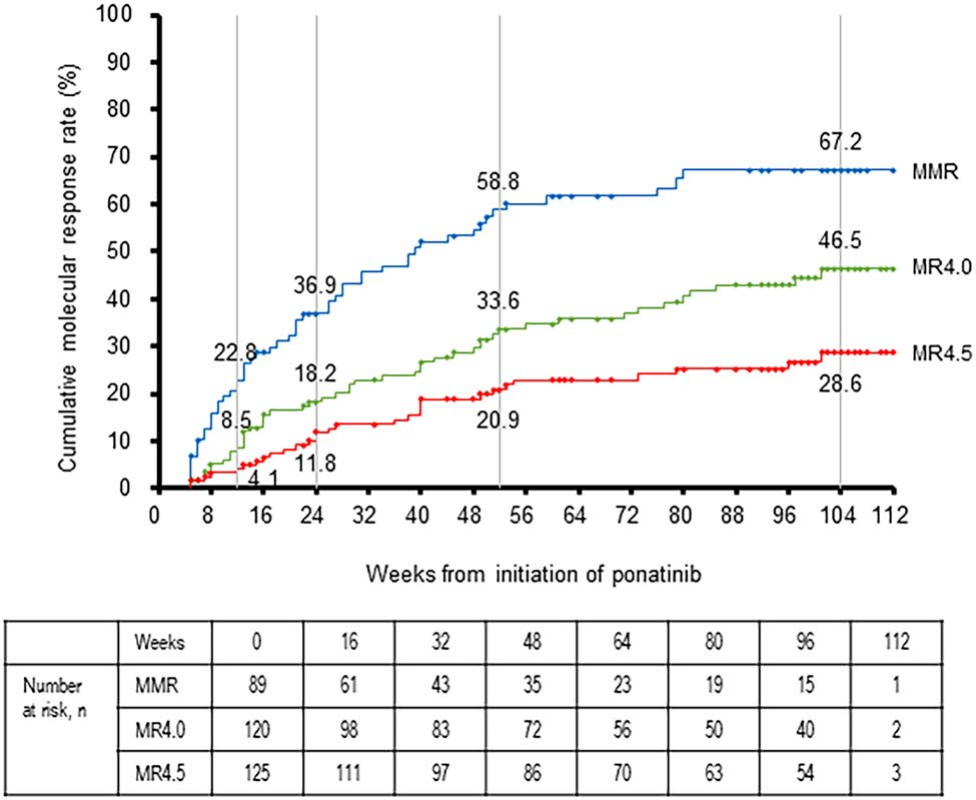
Cumulative molecular response rates in patients with CML-CP who were not achieved major molecular response (MMR) to prior treatment. The cumulative MMR rate, MR4.0 rate and MR4.5 rate in patients with CML-CP patients who did not achieve MMR before treatment with ponatinib are shown. Response rates are labeled at weeks 12, 24, 52, and 104. CML, chronic myeloid leukemia; CP, chronic-phase; IS, international standard; MMR, major molecular response (*BCR::ABL1* transcripts [IS] ≤ 0.1%); MR4.0, molecular response with a 4.0-log reduction (*BCR::ABL1* transcripts [IS] ≤ 0.01%); MR4.5, molecular response with a 4.5-log reduction (*BCR::ABL1* transcripts [IS] ≤ 0.0032%).

Among patients with CML-CP who had not achieved MMR before treatment with ponatinib, the cumulative MMR rate at weeks 12, 24, 52 and 104 was 22.8, 36.9, 58.8 and 67.2%, respectively (Fig. 2). The cumulative rate was 8.5, 18.2, 33.6 and 46.5%, respectively, for MR4.0 and 4.1, 11.8, 20.9 and 28.6%, respectively, for MR4.5.

Among those who were tested for the *BCR::ABL1* mutation and for whom molecular response rate data was available, the cumulative MMR rate at week 52 was 75.3% in patients with the *BCR::ABL1* T315I mutation and 62.0% in patients without the *BCR::ABL1* T315I mutation (Supplementary Fig. 2): the cumulative rate at week 52 was 52.5 and 32.8%, respectively, for MR4.0 and 41.2 and 22.3%, respectively, for MR4.5.

**Figure 3:**
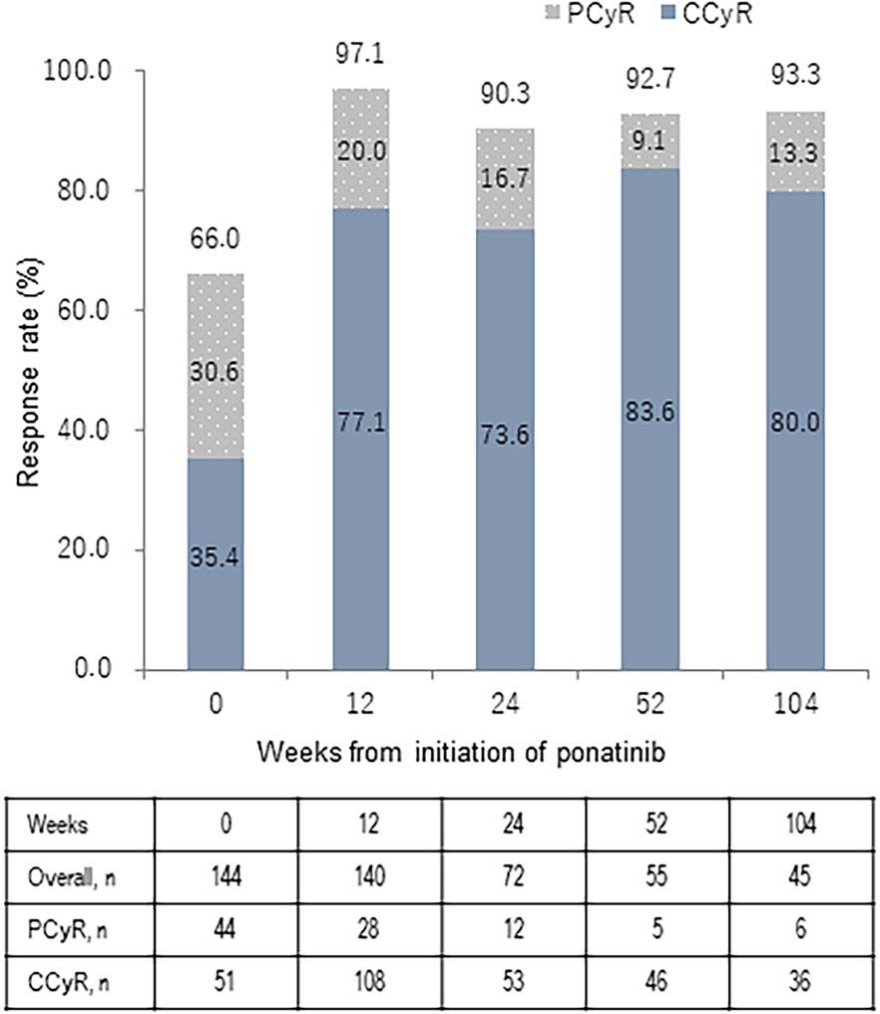
Complete and partial cytogenetic response rates in patients with Ph^+^ ALL. The complete cytogenetic response rate (Solid) and partial cytogenetic response rate (Dotted) before and after initiating ponatinib treatment in patients with Ph^+^ ALL are shown. CCyR, complete cytogenetic response; PCyR, partial cytogenetic response; Ph^+^ ALL, Philadelphia chromosome-positive acute lymphoblastic leukemia.

In patients with Ph^+^ ALL, the CCyR rate at weeks 0, 12, 24, 52 and 104 was 35.4% (51/144), 77.1% (108/140), 73.6% (53/72), 83.6% (46/55) and 80.0% (36/45), respectively (Fig. 3). The MCyR (CCyR and PCyR) rate at weeks 0, 12, 24, 52 and 104 was 66.0% (95/144), 97.1% (136/140), 90.3% (65/72), 92.7% (51/55) and 93.3% (42/45), respectively.

The estimated 1-year overall survival (OS) rate and the estimated 1-year survival rate only considering death due to progressive disease were 98.5 and 100.0%, respectively, in patients with CML-CP (Supplementary Fig. 3A) and 68.6 and 71.2%, respectively, in patients with Ph^+^ ALL (Supplementary Fig. 3B).

## Discussion

In this 2-year post-marketing surveillance of 724 patients with CML or Ph^+^ ALL, ponatinib demonstrated favorable efficacy and tolerability compared with previous clinical trials and also showed the necessity of closely monitoring arterial occlusive events in older adults and patients with predisposing factors for atherosclerosis.

As demonstrated in the PACE trial (7) and a meta-analysis (6), AOEs are considered notable safety risks of ponatinib that require further investigation. In the current surveillance, the incidence of AOEs was 6.49% (47/724), and the exposure-adjusted incidence rate of AOEs per 100 person-years was 6.8, which did not exceed 25% and 13.8 in the PACE trial (7). Among patients with CML-CP in the current surveillance, the incidence of AOEs was 6.22% (12/193) and the exposure-adjusted incidence rate of AOEs per 100 person-years was 4.5, which again did not exceed 31% (84/270) and 14.1 at year 2 in the PACE trial (7).

The most common AOEs in this surveillance were cerebrovascular events in CML-CP and cardiovascular events in Ph^+^ ALL. Although we assume that patients with Ph^+^ ALL were at risk for cardiovascular events because of previous treatment (e.g. anthracycline-based chemotherapy, cyclophosphamide and concomitant glucocorticoids), it is unclear whether AOEs in this surveillance were treatment-related or were due to pre-existing vascular disease. Further studies are necessary to determine whether the type of AOE was affected by the patient’s leukemia type.

The multivariate analyses of pooled data from three clinical trials showed that ponatinib dose intensity, history of ischemic disease and age were strong predictors of increased risk of an AOE, suggesting a 33% reduction in the risk of an AOE or for each 15 mg/day decrease in ponatinib dose intensity (15). The question is whether the dose escalation or reduction method can balance safety and efficacy in patients with highly refractory CML-CP. The phase 2 Optimizing Ponatinib Treatment in CP-CML (OPTIC) trial was conducted to assess the benefit/risk ratio across three ponatinib starting doses (45, 30 and 15 mg/day) in patients with highly resistant CML-CP (16). The results showed that optimal benefit/risk outcomes occurred with a regimen consisting of a 45 mg/day starting dose reduced to 15 mg upon response (*BCR::ABL1* transcripts [IS] ≤ 1%) (16). Furthermore, the 5-year results of the PACE trial, in which patients received an initial dose of 45 mg of ponatinib, showed the exposure-adjusted incidence rate of new AOEs per 100 person-years decreased each year along with median dose intensity among patients with CML-CP, from 15.8/100 person-years at 32.1 mg/day during year 1 to 4.9/100 person-years at 20.4 mg/day during year 4 (7). In the current surveillance, while the maximum daily dose was 45 mg in 36.3% (263/724) of patients, only 19.8% (143/724) of patients received an initial daily dose of 45 mg. Further studies are necessary to ascertain the optimal starting dose to obtain maximum safety and effectiveness in Japanese patients.

Our surveillance showed a significant association between AOEs and age (OR 1.039, 95% CI 1.017–1.062), hypertension (OR 2.137, 95% CI 1.169–3.907) and diabetes (OR 1.950, 95% CI 1.026–3.707). Considering that age and a history of ischemic disease were previously reported as prognostic factors for AOEs (15), a benefit–risk evaluation and close monitoring are especially crucial when treating older patients and those with predisposing factors for atherosclerosis.

The MMR (*BCR::ABL1* transcripts [IS] ≤ 0.1%) rate at month 12 and month 24 was 28 and 34%, respectively, in the PACE trial and 20% and 34%, respectively, in the OPTIC trial (in the group on a 45 mg/day starting dose) (17). In the current surveillance, the cumulative MMR rate in patients with CML-CP who had not achieved MMR before treatment with ponatinib was 58.8% at week 52 and 67.2% at week 104; the cumulative molecular rates (MMR, MR4.0 and MR4.5) increased over time regardless of T315I mutation status, as in the PACE and OPTIC trials. While we cannot directly compare those trials and the current surveillance because of different study designs, the results showed that ponatinib has real-world effectiveness among Japanese patients who were refractory to prior TKIs.

In this surveillance, ponatinib showed a good proportion of complete hematologic remission (73.6%, 237/322) and CCyR (79.7%, 141/177) at any time in Japanese patients with Ph^+^ ALL (data not shown). Furthermore, the estimated 1-year OS rate was 68.6% for Ph^+^ ALL patients and 98.5% for CML patients, which are favorable results and similar to those obtained in the OPTIC and PACE trials (7,16). Taken together, ponatinib has a favorable safety and efficacy profile in Japanese patients with Ph^+^ ALL as well as CML-CP.

This surveillance has some limitations. First, the incidence of AEs in post-marketing surveillance studies may be underreported, especially for lower-grade events. Therefore, we did not statistically compare the incidence of AEs and ADRs in our surveillance to that of previous clinical trials. Second, the prognosis beyond 2 years in patients treated with ponatinib remains unknown. Further studies are necessary to evaluate the long-term safety and efficacy, especially among patients with CML-CP.

In conclusion, this 2-year post-marketing surveillance of patients with CML with resistance/intolerance to prior kinase inhibitors or patients with relapsed/refractory Ph^+^ ALL has demonstrated favorable safety and efficacy of ponatinib in clinical practice in Japan. Older patients and those with predisposing factors for AOEs should be closely monitored for possible vascular adverse events.

## Supplementary Material

JJCO-23-0827_Supplementary_Figure_1_hyae061

JJCO-23-0827_Supplementary_Figure_2_hyae061

JJCO-23-0827_Supplementary_Figure_3_hyae061

JJCO-23-0827_supple_table_hyae061
