# Supplementary figures and images for: Real-world outcomes of ponatinib treatment in 724 patients with CML and Ph+ ALL: a post-marketing surveillance study with a special interest in arterial occlusive events in Japan
Source: Jpn J Clin Oncol. 2024 May 15;54(8):930–8. doi: 10.1093/jjco/hyae061 (PMC11322879; doi:10.1093/jjco/hyae061)

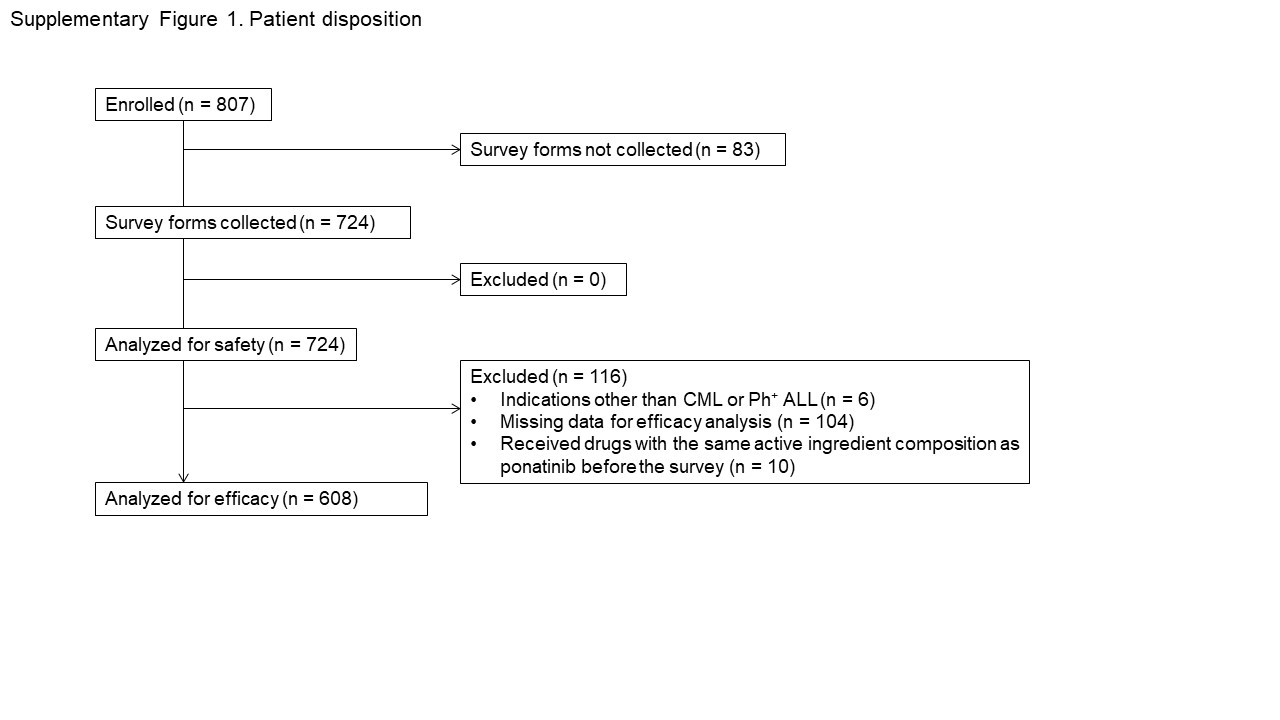

Supplement: JJCO-23-0827_Supplementary_Figure_1_hyae061 [file jjco-23-0827_supplementary_figure_1_hyae061.jpeg]

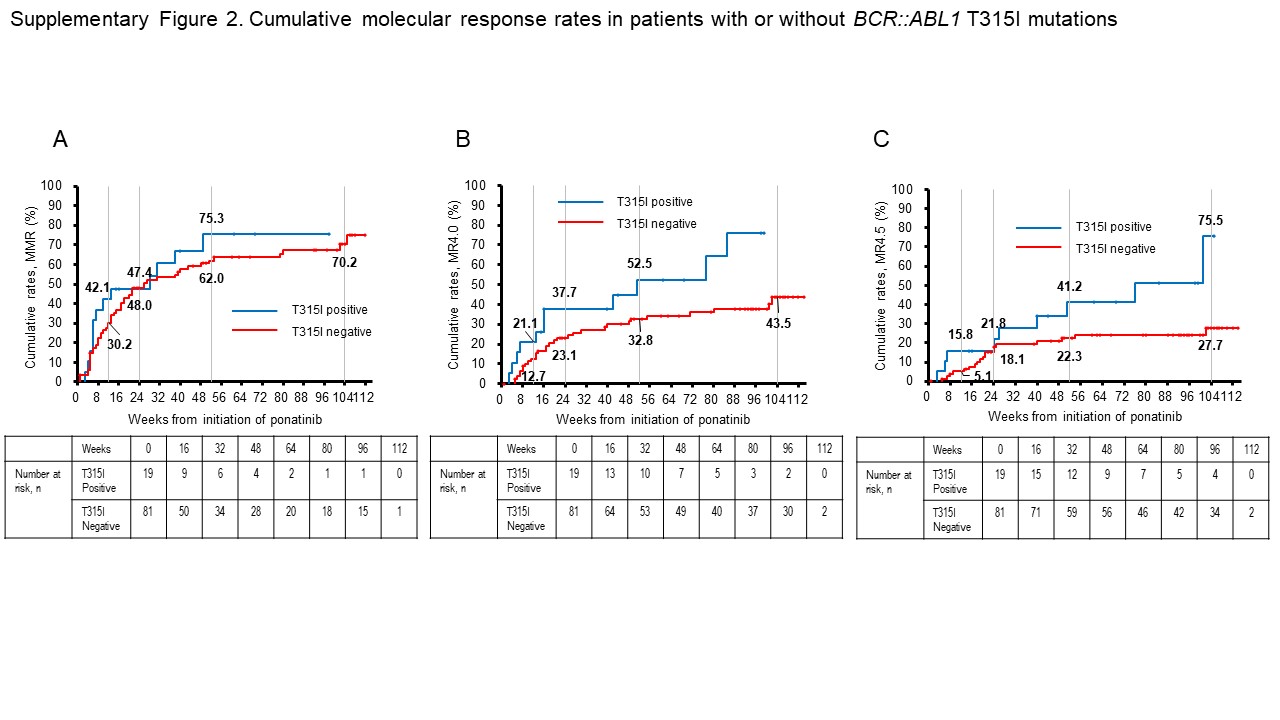

Supplement: JJCO-23-0827_Supplementary_Figure_2_hyae061 [file jjco-23-0827_supplementary_figure_2_hyae061.jpeg]

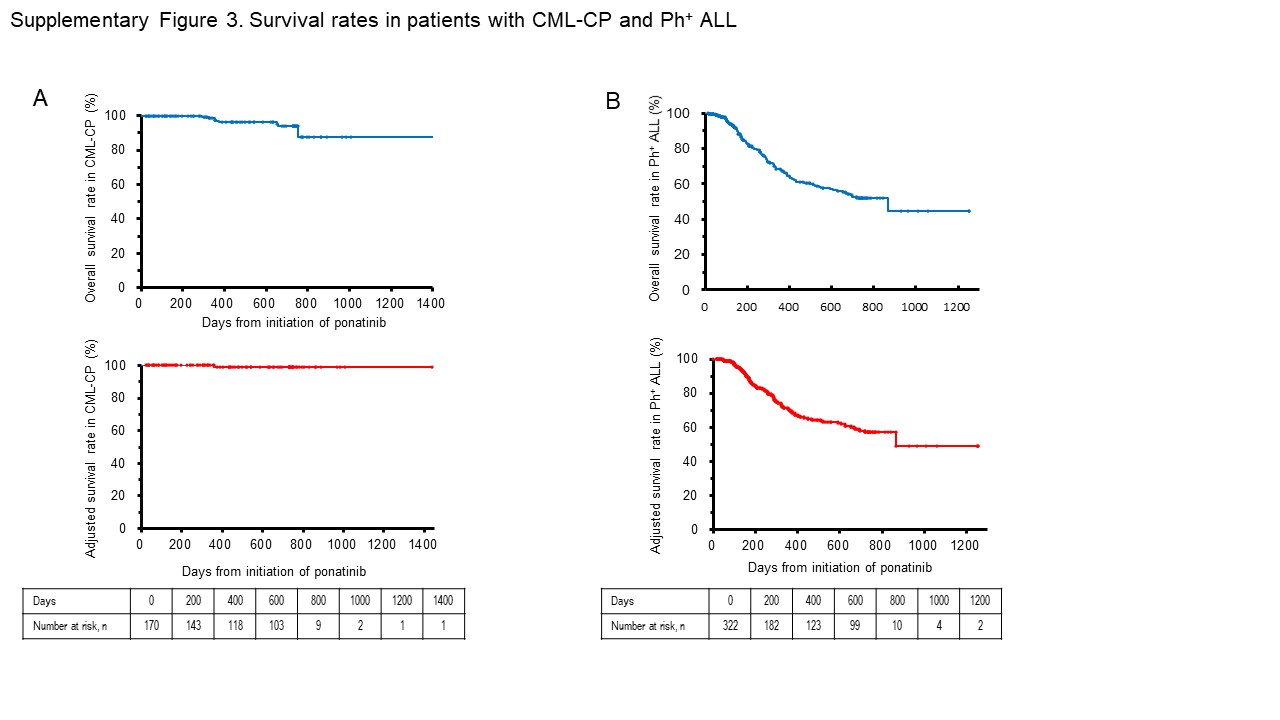

Supplement: JJCO-23-0827_Supplementary_Figure_3_hyae061 [file jjco-23-0827_supplementary_figure_3_hyae061.jpeg]
