# Supplementary material for: Real-world outcomes of ponatinib treatment in 724 patients with CML and Ph+ ALL: a post-marketing surveillance study with a special interest in arterial occlusive events in Japan
Source: Jpn J Clin Oncol. 2024 May 15;54(8):930–8. doi: 10.1093/jjco/hyae061 (PMC11322879; doi:10.1093/jjco/hyae061)
Supplement: JJCO-23-0827_supple_table_hyae061 [file jjco-23-0827_supple_table_hyae061.docx]

Supplementary Table 1. Treatment with ponatinib

|  | Overall | CML-CP | CML-AP | CML-BC | Ph^+^ ALL | Others^b^ |
| --- | --- | --- | --- | --- | --- | --- |
|  | N = 724 | N = 193 | N = 32 | N = 103 | N = 390 | N = 6 |
| Follow-up |  |  |  |  |  |  |
| Median, days | 258.0 | 729.5 | 730.0 | 91.0 | 168.0 | 268.0 |
| Range, days | 1-1436 | 7-1436 | 1-777 | 1-1318 | 1-1254 | 15-731 |
| Not recorded | 1 (0.1) | 1 (0.5) | 0 (0.0) | 0 (0.0) | 0 (0.0) | 0 (0.0) |
|  |  |  |  |  |  |  |
| Starting daily dose |  |  |  |  |  |  |
| < 15 mg | 1 (0.1) | 0 (0.0) | 0 (0.0) | 0 (0.0) | 1 (0.3) | 0 (0.0) |
| 15 mg | 371 (51.2) | 131 (67.9) | 18 (56.3) | 38 (36.9) | 182 (46.7) | 2 (33.3) |
| 30 mg | 207 (28.6) | 39 (20.2) | 8 (25.0) | 34 (33.0) | 125 (32.1) | 1 (16.7) |
| 45 mg | 143 (19.8) | 23 (11.9) | 5 (15.6) | 31 (30.1) | 81 (20.8) | 3 (50.0) |
| > 45 mg | 0 (0.0) | 0 (0.0) | 0 (0.0) | 0 (0.0) | 0 (0.0) | 0 (0.0) |
| Others | 1 (0.1) | 0 (0.0) | 0 (0.0) | 0 (0.0) | 1 (0.3) | 0 (0.0) |
| Not recorded | 1 (0.1) | 0 (0.0) | 1 (3.1) | 0 (0.0) | 0 (0.0) | 0 (0.0) |
|  |  |  |  |  |  |  |
| Maximum daily dose |  |  |  |  |  |  |
| < 15 mg | 0 (0.0) | 0 (0.0) | 0 (0.0) | 0 (0.0) | 0 (0.0) | 0 (0.0) |
| 15 mg | 173 (23.9) | 68 (35.2) | 6 (18.8) | 14 (13.6) | 84 (21.5) | 1 (16.7) |
| 30 mg | 282 (39.0) | 81 (42.0) | 12 (37.5) | 37 (35.9) | 151 (38.7) | 1 (16.7) |
| 45 mg | 263 (36.3) | 42 (21.8) | 12 (37.5) | 52 (50.5) | 153 (39.2) | 4 (66.7) |
| > 45 mg | 0 (0.0) | 0 (0.0) | 0 (0.0) | 0 (0.0) | 0 (0.0) | 0 (0.0) |
| Others | 3 (0.4) | 1 (0.5) | 0 (0.0) | 0 (0.0) | 2 (0.5) | 0 (0.0) |
| Not recorded | 3 (0.4) | 1 (0.5) | 2 (6.3) | 0 (0.0) | 0 (0.0) | 0 (0.0) |
|  |  |  |  |  |  |  |
| Average daily dose^a^ |  |  |  |  |  |  |
| < 15 mg | 116 (16.0) | 36 (18.7) | 4 (12.5) | 10 (9.7) | 64 (16.4) | 2 (33.3) |
| 15 to < 25 mg | 278 (38.4) | 72 (37.3) | 11 (34.4) | 31 (30.1) | 162 (41.5) | 2 (33.3) |
| 25 to < 35 mg | 214 (29.6) | 60 (31.1) | 11 (34.4) | 31 (30.1) | 111 (28.5) | 1 (16.7) |
| 35 to < 45 mg | 61 (8.4) | 14 (7.3) | 4 (12.5) | 13 (12.6) | 30 (7.7) | 0 (0.0) |
| 45 mg | 50 (6.9) | 9 (4.7) | 0 (0.0) | 17 (16.5) | 23 (5.9) | 1 (16.7) |
| > 45 mg | 0 (0.0) | 0 (0.0) | 0 (0.0) | 0 (0.0) | 0 (0.0) | 0 (0.0) |
| Not recorded | 5 (0.7) | 2 (1.0) | 2 (6.3) | 1 (1.0) | 0 (0.0) | 0 (0.0) |
|  |  |  |  |  |  |  |
| Total amount of ponatinib |  |  |  |  |  |  |
| 0 to < 5 g | 355 (49.0) | 51 (26.4) | 12 (37.5) | 71 (68.9) | 216 (55.4) | 5 (83.3) |
| 5 to < 10 g | 117 (16.2) | 34 (17.6) | 4 (12.5) | 10 (9.7) | 69 (17.7) | 0 (0.0) |
| 10 to < 15 g | 123 (17.0) | 45 (23.3) | 4 (12.5) | 10 (9.7) | 63 (16.2) | 1 (16.7) |
| 15 to < 20 g | 50 (6.9) | 23 (11.9) | 3 (9.4) | 6 (5.8) | 18 (4.6) | 0 (0.0) |
| 20 to < 25 g | 44 (6.1) | 23 (11.9) | 5 (15.6) | 2 (1.9) | 14 (3.6) | 0 (0.0) |
| ≥ 25 g | 30 (4.1) | 15 (7.8) | 2 (6.3) | 3 (2.9) | 10 (2.6) | 0 (0.0) |
| Not recorded | 5 (0.7) | 2 (1.0) | 2 (6.3) | 1 (1.0) | 0 (0.0) | 0 (0.0) |

Values are given as n (%).

^a^Average daily dose was calculated by dividing the total amount of ponatinib prescribed over the period by the study duration.

^b^Others include Ph+ ALL intolerance to prior treatment (n = 1), acute myeloid leukemia with c-Kit mutations (n = 1), CML-CP without resistance/intolerance to prior TKIs (n = 1), Ph+ ALL without relapse or refractory disease (n = 2), Philadelphia chromosome-like acute lymphoblastic leukemia (n = 1).

AP, accelerated-phase; BC, blast crisis; CML, chronic myeloid leukemia; CP, chronic-phase; Ph^+^ ALL, Philadelphia chromosome-positive acute lymphoblastic leukemia; TKI, tyrosine kinase inhibitor

Supplementary Table 2. Risks associated with adverse drug reactions, univariable logistic analysis

|  | Adverse drug reactions | |
| --- | --- | --- |
| Factors | Odds ratio | 95% CI |
| Female vs. male | 1.189 | 0.880-1.606 |
| Age | 1.017 | 1.008-1.026* |
| Performance status | 1.139 | 0.963-1.346 |
| Philadelphia chromosome | 1.404 | 0.659-2.991 |
| *BCR::ABL1* transcripts | 0.552 | 0.212-1.441 |
| *BCR::ABL1* mutations | 1.437 | 0.978-2.111 |
| Smoking | 1.103 | 0.785-1.549 |
| Comorbidities |  |  |
| Hypertension | 1.701 | 1.203-2.403* |
| Diabetes | 1.478 | 1.012-2.160* |
| Dyslipidemia | 2.322 | 1.548-3.481* |
| Ischemic disease^a^ | 1.886 | 1.018-3.495* |
| Non-ischemic heart disease | 1.445 | 0.640-3.263 |
| History of ischemic disease^a^ | 1.672 | 1.056-2.647* |
| Average daily dose of ponatinib^b^ | 0.974 | 0.960-0.988* |
| Starting daily dose of ponatinib | 0.998 | 0.985-1.010 |
| Maximum daily dose of ponatinib | 1.003 | 0.990-1.016 |

^a^Ischemic disease includes coronary artery disease, cerebrovascular disease, retinal artery occlusion, peripheral arterial occlusive disease, and venous thromboembolism.

^b^Average daily dose was calculated by dividing the total mass of ponatinib prescribed over the period by the study duration.

*95% CI of the corresponding odds ratio does not include 1.

Supplementary Table 3. Cross intolerance between prior TKIs and ponatinib

|  | Overall | Any CML | CML-CP | CML-AP | CML-BC | Ph^+^ ALL | Others^a^ |
| --- | --- | --- | --- | --- | --- | --- | --- |
|  | N = 724 | N = 328 | N = 193 | N = 32 | N = 103 | N = 390 | N = 6 |
| Prior TKIs |  |  |  |  |  |  |  |
| Imatinib | 278 (38.4) | 140 (42.7) | 93 (48.2) | 16 (50.0) | 31 (30.1) | 138 (35.4) | 0 (0.0) |
| Dasatinib | 640 (88.4) | 287 (87.5) | 166 (86.0) | 28 (87.5) | 93 (90.3) | 348 (89.2) | 5 (83.3) |
| Nilotinib | 174 (24.0) | 160 (48.8) | 115 (59.6) | 20 (62.5) | 25 (24.3) | 14 (3.6) | 0 (0.0) |
| Bosutinib | 134 (18.5) | 131 (39.9) | 88 (45.6) | 17 (53.1) | 26 (25.2) | 3 (0.8) | 0 (0.0) |
| Ponatinib (pre-market use) | 8 (1.1) | 7 (2.1) | 7 (3.6) | 0 (0.0) | 0 (0.0) | 1 (0.3) | 0 (0.0) |
|  |  |  |  |  |  |  |  |
| Intolerance to prior TKIs |  |  |  |  |  |  |  |
| Imatinib | 18 (2.5) | 9 (2.7) | 6 (3.1) | 1 (3.1) | 2 (1.9) | 9 (2.3) | 0 (0.0) |
| Dasatinib | 90 (12.4) | 40 (12.2) | 27 (14.0) | 3 (9.4) | 10 (9.7) | 49 (12.6) | 1 (16.7) |
| Nilotinib | 10 (1.4) | 9 (2.7) | 8 (4.1) | 0 (0.0) | 1 (1.0) | 1 (0.3) | 0 (0.0) |
| Bosutinib | 29 (4.0) | 28 (8.5) | 21 (10.9) | 3 (9.4) | 4 (3.9) | 1 (0.3) | 0 (0.0) |
|  |  |  |  |  |  |  |  |
| Cross intolerance |  |  |  |  |  |  |  |
| Imatinib |  |  |  |  |  |  |  |
| Platelet count decreased | 1 (0.1) | 1 (0.3) | 1 (0.5) | 0 (0.0) | 0 (0.0) | 0 (0.0) | 0 (0.0) |
| Dasatinib |  |  |  |  |  |  |  |
| Lipase increased | 1 (0.1) | 0 (0.0) | 0 (0.0) | 0 (0.0) | 0 (0.0) | 1 (0.3) | 0 (0.0) |
| Platelet count decreased | 2 (0.3) | 2 (0.6) | 2 (1.0) | 0 (0.0) | 0 (0.0) | 0 (0.0) | 0 (0.0) |
| Pleural effusion | 1 (0.1) | 1 (0.3) | 1 (0.5) | 0 (0.0) | 0 (0.0) | 0 (0.0) | 0 (0.0) |
| Nilotinib |  |  |  |  |  |  |  |
| Rash | 1 (0.1) | 1 (0.3) | 1 (0.5) | 0 (0.0) | 0 (0.0) | 0 (0.0) | 0 (0.0) |
| Bosutinib |  |  |  |  |  |  |  |
| Hepatic function abnormal | 1 (0.1) | 1 (0.3) | 1 (0.5) | 0 (0.0) | 0 (0.0) | 0 (0.0) | 0 (0.0) |
| Leukoderma | 1 (0.1) | 1 (0.3) | 1 (0.5) | 0 (0.0) | 0 (0.0) | 0 (0.0) | 0 (0.0) |
| Nausea | 1 (0.1) | 1 (0.3) | 1 (0.5) | 0 (0.0) | 0 (0.0) | 0 (0.0) | 0 (0.0) |
| Pancytopenia | 1 (0.1) | 1 (0.3) | 1 (0.5) | 0 (0.0) | 0 (0.0) | 0 (0.0) | 0 (0.0) |
| Rash | 1 (0.1) | 1 (0.3) | 1 (0.5) | 0 (0.0) | 0 (0.0) | 0 (0.0) | 0 (0.0) |

Values are given as n (%). Some adverse events were attributed to multiple prior TKIs.

^a^Others include Ph+ ALL intolerance to prior treatment (n = 1), acute myeloid leukemia with c-Kit mutations (n = 1), CML-CP without resistance/intolerance to prior TKIs (n = 1), Ph+ ALL without relapse or refractory disease (n = 2), Philadelphia chromosome-like acute lymphoblastic leukemia (n = 1).

AP, accelerated-phase; BC, blast crisis; CML, chronic myeloid leukemia; CML-CP, chronic-phase chronic myeloid leukemia; Ph^+^ ALL, Philadelphia chromosome-positive acute lymphoblastic leukemia; TKI, tyrosine kinase inhibitor

Supplementary Table 4. Incidence of arterial occlusive events^a^

|  | Overall | CML-CP | CML-AP | CML-BC | Ph^+^ ALL | Others^c^ |
| --- | --- | --- | --- | --- | --- | --- |
|  | N = 724 | N = 193 | N = 32 | N = 103 | N = 390 | N = 6 |
| Person-years^b^ | 691.6 | 268.8 | 35.5 | 58.4 | 324.1 | 4.9 |
| Median treatment duration, days | 266.0 | 546.5 | 730.0 | 205.0 | 172.0 | - |
| Median time to event onset, days | 172.5 | 288.0 | 254.0 | 108.0 | 151.5 | - |
| Patients with events, n (%) | 47 (6.49) | 12 (6.22) | 5 (15.63) | 7 (6.80) | 23 (5.90) | 0 (0.00) |
| Incidence, /100 person-years | 6.8 | 4.5 | 14.1 | 12.0 | 7.1 | 0.0 |

Values are given as n (%).

^a^Details of arterial occlusive events are shown in Table 3.

^b^Patients were removed from the calculation when they developed their first arterial occlusive events.

^c^Others include Ph^+^ ALL intolerance to prior treatment (n = 1), acute myeloid leukemia with c-Kit mutations (n = 1), CML-CP without resistance/intolerance to prior TKIs (n = 1), Ph^+^ ALL without relapse or refractory disease (n = 2), Philadelphia chromosome-like acute lymphoblastic leukemia (n = 1).

AP, accelerated-phase; BC, blast crisis; CML, chronic myeloid leukemia; CP, chronic-phase; Ph^+^ ALL, Philadelphia chromosome-positive acute lymphoblastic leukemia
